# Unexpected words that become your best memories: How sentential constraint and word expectedness affect memory retrieval

**DOI:** 10.3389/fnhum.2025.1645907

**Published:** 2025-11-11

**Authors:** Gerrit Höltje, Regine Bader, Julia A. Meßmer, Doruntinë Zogaj, Axel Mecklinger

**Affiliations:** Experimental Neuropsychology Unit, Department of Psychology, Saarland University, Saarbrücken, Germany

**Keywords:** contextual constraint, event-related potentials (ERPs), episodic memory retrieval, predictive language processing, false recognition, familiarity and recollection, inhibitory control, recall-to-reject strategy

## Abstract

Much is known about how the strength of contextual support from strongly constraining (SC) and weakly constraining (WC) sentences influences the online processing of expected (EXP) and unexpected (UNEXP) sentence-ending words. In the present study, we investigated the long-term mnemonic consequences associated with the processing of contextually constraint words and used event-related potentials (ERPs) to explore the memory retrieval mechanisms at work. Furthermore, we investigated false memories for expected but unpresented words. If these unpresented words remained highly accessible in memory, their false recognition as familiar would manifest in a larger early frontal old/new effect, the putative ERP correlate of episodic familiarity. Behavioral results indicated that strongly expected and highly unexpected words were more likely to be recognized, whereas memory for moderately expected words was attenuated. However, the anticipated early frontal old/new effects in these conditions did not materialize. Instead, the retrieval of highly unexpected (SC-UNEXP) words was characterized by a late parietal old/new effect, reflecting a reliance on recollection-based processes. Unexpectedly, during retrieval SC-UNEXP words also evoked a late frontal positivity, a pattern usually associated with the inhibition of unpresented expected words during encoding. This suggests that the retrieval of these words reactivated inhibitory mechanisms akin to those activated during encoding. Additionally, expected lures that were correctly identified as new elicited a broadly distributed positive slow wave, indicative of recollective processing in support of a recall-to-reject strategy. This latter effect was observed irrespective of the predictive strength of the contextual support.

## 1 Introduction

Learning is most effective when new information can be integrated into an existing schema–an associative knowledge structure formed through repeated experiences (Alba and Hasher, 1983; Bartlett, 1932; Bransford and Johnson, 1972; Hebscher et al., 2019). Schemas can be activated by contextual cues and help to predict future events that have previously been linked to similar contexts (Ghosh and Gilboa, 2014). The sentence context “She went to the bathroom and cleaned her teeth with a,” for example, could activate the reader’s bathroom schema, enabling the prediction of the word “toothbrush” as the most likely completion. Activated schemas, as the bathroom schema in the example above, are believed to enhance the encoding of congruent or expected information (“toothbrush”), fostering robust and easily retrievable memory representations (Craik and Tulving, 1975; Greve et al., 2019; Staresina et al., 2009). Thereby, contextually expected words like “toothbrush” should be better remembered than a less predictable word like “toothpick,” which does not violate the bathroom schema, but is also not part of it.

Unexpected information that violates the prediction supported by the schema is remembered better than unrelated (neutral) information. However, the underlying mechanisms are different than for schema-congruent encoding. While words like “toothpick,” which are less expected than “toothbrush,” remain congruent with the aforementioned sentence context, yet they elicit an expectancy mismatch that activates schema accommodation and assimilation processes (Ghosh and Gilboa, 2014; Gilboa and Marlatte, 2017; Piaget, 1952). Notably, the integration of unexpected but plausible words into an activated schema may require the inhibition of more expected words (Kutas, 1993; Ness and Meltzer-Asscher, 2018a; Van Petten and Luka, 2012). Expectancy mismatch processing aims to reduce future prediction errors, potentially by enhancing memory for the unexpected event (Friston, 2010; Henson and Gagnepain, 2010). Recent studies provide behavioral evidence supporting a prediction error-related memory enhancement. Furthermore, there is strong empirical evidence linking the encoding of surprising events to hippocampal processing (for a recent review, see Shing et al., 2023). However, the consequences of prediction-error driven encoding of unexpected information for their subsequent retrieval remain unclear. By investigating neural activity during retrieval, we seek to shed light on the processes involved, providing insights into the neural mechanisms of this type of processing and the quality of the retrieved memory representations. In the present study, we used event-related potentials (ERPs) to unravel the processes involved in the retrieval of contextually expected words (“toothbrush”), unexpected words eliciting expectancy mismatches (“toothpick”), and to compare them with the retrieval of words which are neither strongly expected nor unexpected.

Notably, the effects of schema congruency and prediction errors described above depend on whether sentence contexts provide rich associative connections that activate specific schemas enabling stronger schema-based predictions, i.e., these contexts are strongly constraining in which word comes next. Clearer predictions should in turn facilitate the processing of expected words. For example, a strongly constraining context like “He locked the door with the” activates relevant conceptual information, enabling the prediction of words like “key.” In contrast, a weakly constraining context like “For lunch, he had” does not support strong predictions (Piai et al., 2016). In a previous study (Höltje and Mecklinger, 2022), we used sentence contexts that were either strongly constraining (SC: “In this heat the flower urgently needs more…”) or weakly constraining (WC: “Before turning in his bachelor’s thesis, Luke makes an appointment with his…”) to examine how context strength modulates the encoding of expected words that confirm predictions and unexpected words that violate them. Event-related potentials (ERPs) were recorded while participants read the sentences. Our findings showed that better memory performance for expected words compared to unexpected ones was accompanied by a parietal subsequent memory effect (SME), an ERP effect that is usually elicited when an item-specific memory trace for a study event is generated (Mecklinger and Kamp, 2023).

However, contrary to our expectations, we did not find a beneficial effect of prediction error on memory, as unexpected words were remembered worse than expected words. In addition, ERPs did not show evidence of expectancy mismatch-related processing during encoding that would predict successful recognition of unexpected words a day later. A possible explanation for the absence of a prediction error effect on memory in our study may be that memory for unexpected words decreased over the 24-h retention delay between study and test, while memory for contextually expected words remained stable. In support of this view, recent research suggests that schema effects on memory often become more pronounced after a 1-day retention interval, likely due to the accelerated consolidation of schema-congruent information (van Kesteren et al., 2013). In our study, any potential memory benefits from prediction errors may have been diminished by the end of the 24-h retention delay, due to sleep-associated decay of hippocampal memory traces (e.g., Hardt et al., 2013), against which schema-based memory effects are more protected due to their accelerated consolidation.

In the present study, our goal was to examine how the strength of schema support provided by sentence contexts affects the retrieval of expected words that confirm predictions and unexpected words that trigger expectancy mismatches, and how these processes are reflected in ERP measures during retrieval. During the learning phase, participants read sentences that were either strongly constraining or weakly constraining regarding the sentence-ending word (SC: “In this heat the flower urgently needs more…”; WC: “Before turning in his bachelor’s thesis, Luke makes an appointment with his…”). The sentences ended either with highly expected words (SC: “water”; WC: “professor”) or unexpected but contextually congruent words (SC: “protection”; WC: “advisor”). In an ensuing surprise recognition memory test, participants were asked to discriminate between target words from the learning phase and unrelated new words. EEG recordings during retrieval allowed us to compare studied words correctly identified as “old” (hits) with new words correctly identified as “new” (correct rejections). This design enabled us to assess the mnemonic effects and ERP measures during retrieval linked to confirmed predictions (expected words) and expectancy mismatches (unexpected words).

Building on schema theory and our prior findings, we hypothesized that highly predictive SC sentences would provide strong schema support, enhancing memory performance for expected words. In contrast, highly unexpected words eliciting expectancy mismatches should also be better remembered than moderately expected words, i.e., words in weakly constraining sentences. Consequently, the relationship between word expectedness and memory is expected to follow a U-shaped pattern (Brod et al., 2022; Greve et al., 2019; Quent et al., 2022; Shing et al., 2023; van Kesteren et al., 2012). To allow for better comparability of our results with the results of these previous studies, we reduced the retention interval of the current study to 12 min and assumed that this would result in a memory enhancing effect of prediction error during online language processing.

Examining neural activity during the retrieval of studied words can provide insights into the processes, neural mechanisms, and quality of retrieved memory representations. Therefore, this study investigated the long-term effects of predictive language processing on memory retrieval by comparing ERP old/new effects during a recognition memory test. We add to few existing ERP studies (Hubbard et al., 2019; Hubbard and Federmeier, 2024; Rommers and Federmeier, 2018) in adopting a dual-process model of recognition memory approach (Yonelinas, 2002) that has, to our knowledge, not yet been used in the context of predictive language processing (but see Hubbard and Federmeier, 2024, for an investigation of N400 and LPC effects). We examined early frontal old/new effects and later parietal old/new effects as ERP correlates of episodic familiarity and recollection (see Mecklinger and Bader, 2020, for a recent review).

If strongly constraining sentence contexts enhance the encoding of expected words by increasing their semantic activation or integration (Hubbard et al., 2019), their retrieval should rely more on relative familiarity, reflected in a larger early frontal old/new effect on SC-EXP as compared to WC-EXP words. In addition, if these contexts foster strong associations with expected words during encoding, their retrieval should involve recollection of contextual details, resulting in a larger late parietal old/new effect for SC-EXP versus WC-EXP words. On the other hand, if unexpected words create expectancy mismatches that improve memory encoding through increased hippocampal processing, their retrieval should rely on recollective processing and give rise to more pronounced late parietal old/new effects for SC-UNEXP words as compared to WC-UNEXP words that are neither highly expected nor highly unexpected.

Beyond investigating schema effects on memory retrieval for sentence-ending words, this study also aimed to examine the fate of expected but unseen words in memory. Recent research suggests that words predicted by context, even if not presented, can remain accessible in memory and lead to false memory decisions in ensuing tests of long-term memory (Höltje and Mecklinger, 2022; Hubbard et al., 2019; Rich and Harris, 2021; Rommers and Federmeier, 2018). In a study by Hubbard et al. (2019), participants read sentences with either expected or unexpected endings, and later their recognition memory was tested for the sentence-ending words, expected but unpresented words (expected lures), and new words. The results showed that highly predictable but unpresented words (expected lures) were more likely to produce false positive memory decisions than unrelated new words. This suggests that predictive processing triggered by a sentence context can lead to mnemonic costs when a word does not match the predicted word, possibly due to the pre-activation of the predicted word in memory.

If expected but unpresented words remain in a heightened state of activation, they may be processed with greater fluency in the ensuing test phase, leading to false positive memory decisions. Our previous study found that strongly expected lures led to more false positives than less expected lures even 24 h after the encoding phase, and less expected lures were still associated with more false alarms than entirely new words (Höltje and Mecklinger, 2022). The present study investigated whether false positive memory decisions for expected but unpresented words are associated with increased processing fluency, using ERPs to assess the quality of these false memories. Behaviorally, we expected to replicate the pattern of false alarm rates from our previous study: SC lures > WC lures > new words. If expected lures induce fluency that biases memory judgments, they should elicit early frontal old/new effects. Moreover, if processing fluency underlies the false alarm effect in memory, these early frontal old/new effects should be stronger for strongly constraining (SC) lures than for weakly constraining (WC) lures.

## 2 Materials and methods

### 2.1 Participants

Thirty-eight young adults, all native German speakers and right-handed as affirmed by the Edinburgh Handedness Inventory (Oldfield, 1971), partook in the study. They possessed normal or corrected-to-normal vision and reported no neurological or psychiatric conditions. The experimental protocols obtained approval from the ethics board of the Faculty of Human and Business Sciences at Saarland University. Prior to commencement, participants provided informed consent. They received compensation in the form of either €10 per hour or course credit. Data from two participants were excluded from all behavioral analyses because their memory performance did not significantly exceed chance level, as determined by individual binomial tests on trial accuracies during the test phase. Thus, behavioral analyses were conducted on the dataset comprising *N* = 36 participants, *n* = 28 of whom were female, with ages spanning from 18 to 32 years and a median age of 22 years. However, due to exclusion criteria concerning ERP data, those analyses are based on a reduced number of datasets (see Section “2.5 ERP analyses”).

The sample size was determined by considering the smallest behavioral effect identified in our previous study [i.e., the main effect of Constraint in Höltje and Mecklinger (2022)]. Using R (R Core Team, 2024) and RStudio (RStudio Team, 2020), we calculated the sample size required for a one-way repeated measures ANOVA with two levels. With parameters set to *f* = 0.47, α = 0.05, 1−β = 0.80, and employing two-sided testing, we arrived at a sample size of *N* = 38 participants for this investigation.

### 2.2 Stimuli

In the experiment, a total of 200 sentence frames were utilized, half of which were strongly constraining (SC) regarding the final word. Constraint was determined through cloze probabilities obtained in a separate norming study detailed in our prior work (Höltje and Mecklinger, 2022). The remaining frames were weakly constraining (WC), lacking a specific expectation for the final word. In this study, cloze probabilities for expected target words were ≥0.60 (*M* = 0.86, SD = 0.12) for SC frames and ≤0.45 (*M* = 0.26, SD = 0.09) for WC frames. Sentence lengths were matched between the two constraint types.

During the study phase, half of the sentences were completed with expected target words having high cloze probabilities, while the other half was completed with unexpected target words having near-zero cloze probabilities. This resulted in four experimental conditions: SC frames with expected targets (SC-EXP), SC frames with unexpected targets (SC-UNEXP), WC frames with expected targets (WC-EXP), and WC frames with unexpected targets (WC-UNEXP).

All target words, singular nouns, were matched for word length (SC-EXP: *M* = 6.70, SD = 2.65; SC-UNEXP: *M* = 6.52, SD = 2.57; WC-EXP: *M* = 6.62, SD = 2.48; WC-UNEXP: *M* = 6.64, SD = 2.76) and frequency (SC-EXP: *M* = 53.14, SD = 108.60; SC-UNEXP: *M* = 47.11, SD = 82.01; WC-EXP: *M* = 55.35, SD = 109.06; WC-UNEXP: *M* = 47.15, SD = 72.08) using normalized lemma frequencies from the dlexDB database (Heister et al., 2011). Additionally, 150 singular nouns, matched in word length (*M* = 6.83, SD = 2.38) and frequency (*M* = 42.35, SD = 78.20), were retrieved from the dlexDB database and presented as new words during the test phase of the experiment. For examples of the stimuli, see Table 1.

**TABLE 1:** Examples of the sentences and words that were used in the experiment.

Constraint condition	Sentence frame	Expected	Unexpected
SC	The physician saved the patient’s	Life	Eye
WC	Anita wants to pay for her purchases, but she does not find her	Wallet	Card
SC	Attentively, Anke searched the grass for a four-leaf	Clover	Daisy
WC	While cleaning up the cellar, Alice found a chest full of	Dust	Gold
SC	After her operation, Susanne gets something against her	Pain	Hunger
WC	Because Sandra has a long-distance relationship, she often flies to	London	Munich
SC	When her fridge is empty, Elsa goes to the	Supermarket	Grandpa
WC	On vacation, Rolf takes a lot of photographs of his	Hotel	Excursion

Please note that minor adaptations were made to the stimuli for the translation from German into English. SC, strong constraint; WC, weak constraint.

### 2.3 Procedure

The experiment was divided into a study phase lasting 30 min and a subsequent test phase lasting 40 min, with a 12-min interval in between. During this interval, participants performed an oddball task unrelated to the present study’s objectives. EEG recording setup took approximately 45 min. Thereafter, participants were seated in front of a screen within an electrically shielded and sound-attenuated booth. Experimental tasks were administered using E-Prime 2.0 software (Psychology Software Tools, Pittsburgh, PA), and participants utilized a keyboard for their responses. List and key assignments were balanced across all participants.

#### 2.3.1 Study phase

Participants underwent a total of 200 trials, distributed evenly across the four experimental conditions (SC-EXP, SC-UNEXP, WC-EXP, WC-UNEXP), with an additional four practice trials. The 200 study trials were organized into five blocks of 40 trials each, interspersed with self-paced breaks. Participants were instructed to carefully read the sentence frames and words. In 25% of all trials, they were required to answer a yes/no comprehension question related to the sentence frame. Trial presentation followed a pseudorandomized order to ensure that no more than three trials of the same experimental condition appeared consecutively and that no more than three successive trials included a comprehension question.

Each trial began with a fixation cross (500 ms), followed by the presentation of a sentence frame (5000 ms) and a blank screen (500 ms). A fixation cross (500 ms) preceded the appearance of the target word (1500 ms), followed by a blank screen (500 ms), and, in one-third of the trials, a comprehension question (self-paced, maximum 5000 ms). Participants responded to the comprehension questions using the “c” and “n” keys on the keyboard. Trials were separated by an inter-trial interval that varied between 1500 and 2000 ms.

To assess participants’ engagement in the task, and to ensure they attended to the sentence content, the proportion of correct responses to comprehension questions was calculated and analyzed.

#### 2.3.2 Test phase

In the surprise recognition memory test, the 200 target words from the study phase were paired with 250 new words. These new words comprised 150 unrelated items and 100 words that were expected but not seen during the study phase (lures). Specifically, for each of the 100 sentence frames completed with an unexpected word during the study phase, the anticipated but unseen word was presented as an expected lure in the test phase. Old and new words were presented in a pseudorandomized order, ensuring that no more than three consecutive target or new/lure items were shown. The 450 test trials were divided into six blocks (five blocks of 80 trials each, and the last block comprising 50 trials) separated by self-paced breaks.

At the start of each trial, a fixation cross (500 ms) was displayed, followed by a word (1000 ms). Participants were instructed to determine whether each word was old or new by pressing the “c” and “n” keys on the keyboard (key assignments were balanced across participants). After the word presentation, a blank screen appeared for 1000 ms. Subsequently, the question “Old or New?” along with a depiction of the response keys was shown. Participants could provide their old/new decision as soon as the word was presented. Following the participant’s response, a blank screen was displayed, jittered between 1500 and 2000 ms, before the next trial commenced. If participants failed to respond within 3 s, a feedback screen indicated that their response was too slow, and data from these trials were excluded from analysis.

Memory performance was assessed by calculating hit and false alarm rates, representing the proportions of correct and incorrect “old” decisions, respectively. These rates were then analyzed based on the experimental conditions.

### 2.4 EEG recording and processing

The EEG was recorded from 28 Ag/AgCl scalp electrodes embedded in an elastic cap with positions according to the 10–20 electrode system (Fp1, Fp2, F7, F3, Fz, F4, F8, FC5, FC3, FCz, FC4, FC6, T7, C3, Cz, C4, T8, CP3, CPz, CP4, P7, P3, Pz, P4, P8, O1, O2, and A2). Vertical and horizontal electrooculograms (EOG) were recorded using four electrodes positioned above and below the right eye and at the canthi of the left and right eyes. The electrodes were online referenced to a left mastoid electrode (A1), with AFz serving as the ground electrode. EEG signals were amplified with a BrainAmp DC amplifier (Brain Products GmbH, Gilching, Germany) within a frequency range of 0.016–250 Hz and digitized at 500 Hz.

For offline processing of the EEG data collected during the test phase, the EEGLAB (Delorme and Makeig, 2004) and ERPLAB (Lopez-Calderon and Luck, 2014) toolboxes in MATLAB (The MathWorks Inc., Natick, MA) were used. Electrodes were re-referenced to the average of the left and right mastoid electrodes. The data underwent bandpass filtering between 0.1 and 30 Hz using a second-order Butterworth filter. Additionally, a Parks-McClellan Notch filter was employed to eliminate line noise at 50 Hz frequency. Segments were extracted from 200 ms before the onset of the target word to 1000 ms thereafter. The segments were baseline-corrected using the activity observed during the 200 ms preceding the target word onset. To address ocular artifacts, independent component analysis was applied to the segmented data. Components associated with ocular artifacts were identified and manually removed based on their activations and topographies. Segments containing artifacts were rejected according to specific criteria, including a minimum and maximum total amplitude of ±80 μV, a maximum difference of 100 μV between values within 200 ms intervals (with window steps of 100 ms), a maximum allowed voltage step of 30 μV/ms, and a flatlining threshold of ±0.6 μV for durations of 200 ms. On average, 3.54% of the segments were rejected.

### 2.5 ERP analyses

Event-related potentials were averaged for hits, which are old words correctly judged as “old,” across the experimental conditions (SC-EXP, SC-UNEXP, WC-EXP, WC-UNEXP). Additionally, ERPs for correct rejections (new words correctly judged as “new”) and false alarms (new words incorrectly judged as “old”) were averaged for new words (NW) and expected lures (SC-L, WC-L), respectively.

For ERP analyses involving hits, two data sets were excluded due to an insufficient number of artifact-free trials (<7) in one of the conditions (for ERP studies using a similar criterion, see Höltje et al., 2019; Höltje and Mecklinger, 2022; Kamp et al., 2018). Consequently, analyses pertaining to hits are based on data from *n* = 36 participants. The mean and range of trial numbers per condition and participant were as follows: *M* = 25, range 10–41 (SC-EXP hits), *M* = 24, range 8–36 (SC-UNEXP hits), *M* = 23, range 8–40 (WC-EXP hits), *M* = 21, range 7–42 (WC-UNEXP hits).

For ERP analyses involving correct rejections (CR) and false alarms (FA), additional eight data sets were excluded due to an insufficient number of artifact-free trials (<7) in one of the conditions. Therefore, analyses concerning CR and FA are based on data from *n* = 28 participants. The mean and range of trial numbers per condition and participant were as follows: *M* = 35, range 18–48 (SC-L CR), *M* = 37, range 15–48 (WC-L CR), *M* = 123, range 44–146 (NW CR), *M* = 14, range 7–27 (SC-L FA), *M* = 13, range 7–23 (WC-L FA).

For the planned analyses, mean amplitudes were assessed within two consecutive time windows. The first window spanned from 300 to 500 ms, targeting early mid-frontal old/new effects (Mecklinger and Bader, 2020). The second window, adjacent to the first, extended from 500 to 800 ms, capturing late parietal old/new effects, which are typically largest during this timeframe (Friedman and Johnson, 2000; Rugg and Curran, 2007).

In order to capture both frontally- and parietally-distributed old/new effects, the electrode montage consisted of 12 electrodes that cover anterior and posterior brain regions, divided into two electrode clusters (anterior: F3, Fz, F4, FC3, FCz, FC4; posterior: CP3, CPz, CP4, P3, Pz, P4).

### 2.6 Statistical analyses

Statistical analyses were performed using IBM SPSS software and R (version 4.4.1; R Core Team, 2024). To analyze the recognition memory performance for both hits and false alarms, we ran generalized mixed-effects models (Jaeger, 2008) using the lme4 package in R (e.g., Bates et al., 2015), predicting whether participants made a correct or incorrect recognition response (0 = incorrect; 1 = correct) on trial-level behavioral data. For target words, fixed effects included Constraint, Expectedness, and their interaction, as well as word length and word frequency to account for lexical variability. The “maximal” model (Barr et al., 2013) included intercepts and slopes for participants for constraint and expectedness. To reduce multicollinearity, categorical predictors were contrast coded (strong = −0.5, weak = 0.5; expected = −0.5, unexpected = 0.5), word length were scaled, and word frequency values were log-transformed and scaled. In cases of non-convergence or singularity, the random-effects structure was simplified following the least-variance approach (Bates et al., 2015). *P*-values (*p* < 0.05) were obtained via Wald tests from model summaries.

Similarly, to test if the number of hits is higher in the strong-constraint expected (SC-EXP) and unexpected (SC-UNEXP) conditions relative to the weak-constraint unexpected (WC-UNEXP) condition, we ran a generalized mixed-effects model with planned contrasts. Specifically, we included only one factor with four levels in the model and contrasts were defined to test (1) SC-EXP vs. WC-UNEXP and (2) SC-UNEXP vs. WC-UNEXP, while WC-UNEXP served as the reference category. This coding allowed us to directly evaluate the U-shaped prediction of schema theory. To examine behavioral false alarm rates, we fit another mixed-effects model with the following two sets of contrasts: (1) lures (collapsed across strong and weak constraints) vs. new words, and (2) strong-constraint vs. weak-constraint lures. These models used the same random-effects structure and covariates described for the target words.

Electrophysiological measures underwent examination via repeated-measures ANOVAs and dependent *t*-tests. In instances where the assumption of sphericity was violated, Greenhouse-Geisser corrected degrees of freedom and *p*-values are reported. Significant effects were further explored through lower level ANOVAs and dependent *t*-tests. Partial eta squared (*η*_p_^2^) was utilized as a measure of effect size for ANOVA results, while Cohen’s *d* was calculated for independent *t*-tests. For dependent *t*-tests, *d* was computed following the method outlined by Dunlap et al. (1996), accounting for correlations between measurements.

## 3 Results

### 3.1 Behavioral results

The study revealed a high proportion of correct responses to comprehension questions during the study phase (*M* = 0.90, SEM = 0.01), indicating participants’ compliance with instructions and their attentiveness to sentence content. Individual binomial tests confirmed that each participant’s accuracy in the responses to the comprehension questions was significantly above chance. During the test phase, *Pr* scores (*M* = 0.28, SEM = 0.02) significantly exceeded zero, *t*(35) = 12.85, *p* < 0.001, *d* = 2.14, suggesting participants effectively distinguished between studied target words and new words. Mean hit rates and false alarm rates for each condition are detailed in Table 2. Log-transformed and scaled word frequency values for each condition: *M* = 1.26, SD = 0.70 (SC-EXP); *M* = 1.18, SD = 0.72 (SC-UNEXP); *M* = 1.25, SD = 0.68 (WC-EXP); *M* = 1.33, SD = 0.59 (WC-UNEXP).

**TABLE 2:** Mean proportions and standard deviations of “old” responses to targets (hit rates), unrelated new words, and lures (false alarm rates) in the memory test.

Condition	“Old” responses
SC-EXP targets	0.50 (0.16)
SC -UNEXP targets	0.50 (0.15)
WC-EXP targets	0.49 (0.17)
WC-UNEXP targets	0.44 (0.17)
SC lures	0.27 (0.11)
WC lures	0.21 (0.10)
Unrelated new words	0.13 (0.06)

The final model for hit rates included fixed effects for Constraint, Expectedness, word length and frequency as well as by-subject random intercepts. This analysis revealed a significant main effect of Constraint, indicating better memory for target words following strongly constraining sentence frames (*M* = 0.50, SEM = 0.02) compared to weakly constraining ones (*M* = 0.46, SEM = 0.03; β = −0.13, *z* = −2.68, *p* < 0.05), and a main effect of word frequency, with lower-frequency words leading to more hits (β = −0.22, *z* = −8.19, *p* < 0.001). However, no other effect reached significance.

Next, to test whether hit rates followed a U-shaped function (van Kesteren et al., 2012), as predicted by schema theory, we compared hit rates in the SC-EXP (most expected) and SC-UNEXP (most unexpected) conditions with those in the WC-UNEXP (neither expected nor unexpected) condition. Both SC-EXP (β = 0.21, *z* = 3.01, *p* < 0.01) and SC-UNEXP (β = 0.22, *z* = 3.11, *p* < 0.01) showed significantly higher hit rates than WC-UNEXP, consistent with a U-shaped schema effect.

The results of false alarm performance showed that expected lures produced more false alarms compared to new words (β = 0.25, *z* = 13.06, *p* < 0.001), and that strong-constraint lures elicited more false alarms than weak-constraint lures (β = 0.17, *z* = 4.20, *p* < 0.001). Lastly, word frequency exerted a strong negative effect (β = −0.37, *z* = −7.71, *p* < 0.001), such that high-frequency words were more likely to be falsely recognized. To summarize, memory for target words was generally better after strongly constraining sentences, especially for low-frequency words. Hit rates were highest for both expected and unexpected words in strong contexts, showing a U-shaped pattern consistent with schema theory. False alarms were more frequent for expected lures and strong-context lures than for new or weak-context lures, with high-frequency words more prone to false recognition.

### 3.2 ERP results

#### 3.2.1 Target words in the four experimental conditions

**FIGURE 1:**
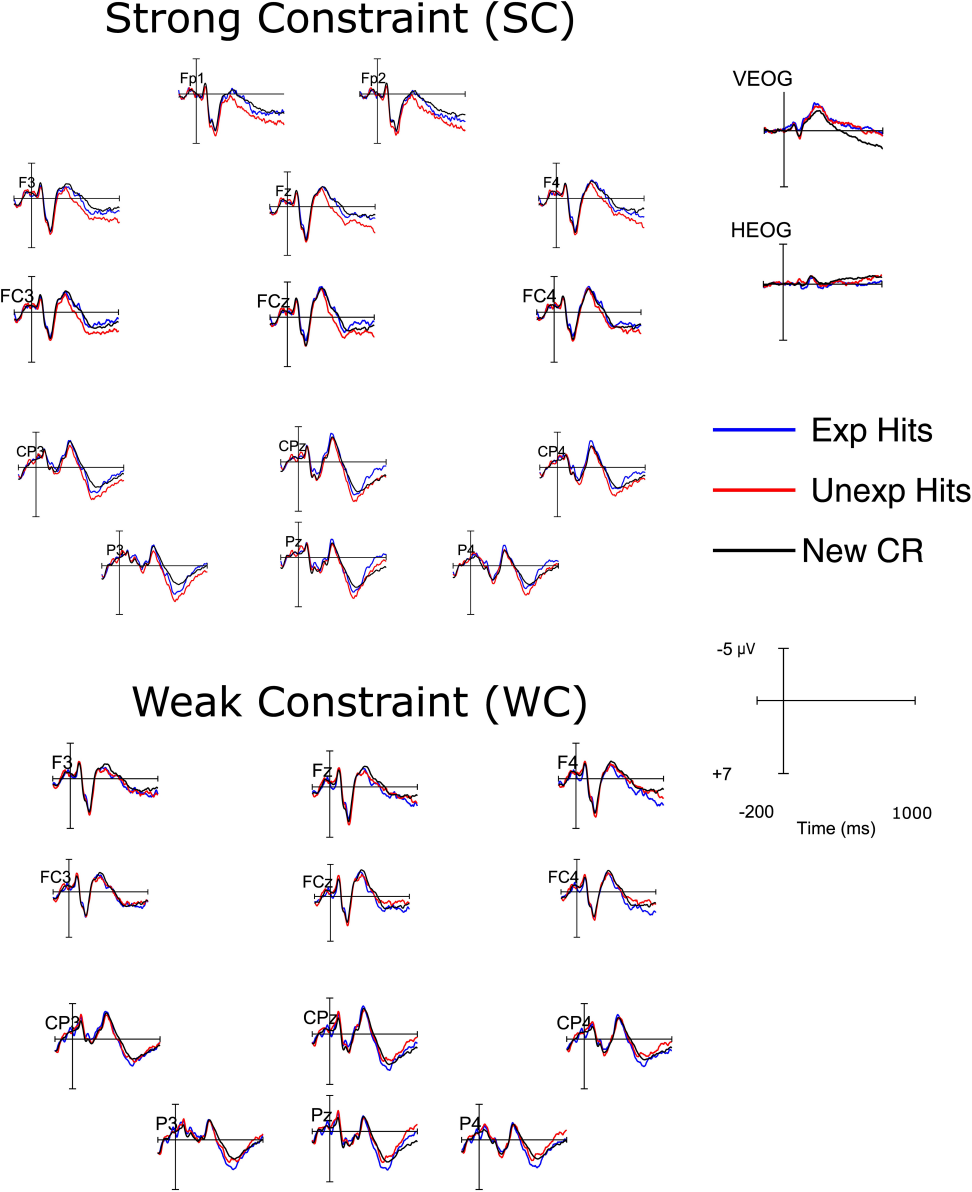
Event-related potentials (ERP) waveforms elicited at electrodes of the anterior (F3, Fz, F4, FC3, FCz, FC4) and posterior (CP3, CPz, CP4, P3, Pz, P4) electrode clusters by the onset of target words in the recognition memory test. In the top half, words encoded in (SC) sentences, prefrontal (Fp1 and Fp2) and (horizontal and vertical) EOG electrodes are included. Bottom half: words encoded in weak constraint (WC) sentences. The alignment of waveforms corresponds to the approximate topographical locations of electrodes over the scalp.

Figure 1 displays ERPs evoked by target words accurately identified as “old” (hits) and new words correctly identified as “new” (correct rejections) during the test phase. Of note, the to-be-rejected new words used in the memory test were independent from the factors Constraint and Expectancy. Because of this, ERPs to new words used to calculate old/new effects are identical across conditions. As a result, differences in old/new effects across conditions can only arise from differences in ERPs to hits across conditions. Therefore, a two-step analysis procedure was performed to test our hypotheses regarding early frontal and late parietal old/new effects: In a first step, dependent *t*-tests were conducted to investigate differences in ERPs to hits between conditions. In a second step, we tested if there were statistically reliable differences between ERPs on hits and correct rejections in each of the four experimental conditions.

For the early frontal old/new effect, mean amplitudes between 300 and 500 ms at anterior electrodes were included in the *t*-test. Unexpectedly, the ERPs to hits did not differ significantly between the SC-EXP (*M* = −2.14 μV, SEM = 0.70) and WC-EXP (*M* = −1.75 μV, SEM = 0.62) conditions, *t*(35) = 0.81, *p* = 0.21 (one-tailed), *d* = 0.10. To assess the significance of the old/new effect in each condition, differences between hits and correct rejections were calculated for each condition and compared against zero using one-sided testing. To address multiple comparisons, the critical *p*-value was adjusted to 0.0125 (0.05/4), using the Bonferroni correction. Old/new effects in this time window were significant in none of the conditions (all *p*-values > 0.03).

**FIGURE 2:**
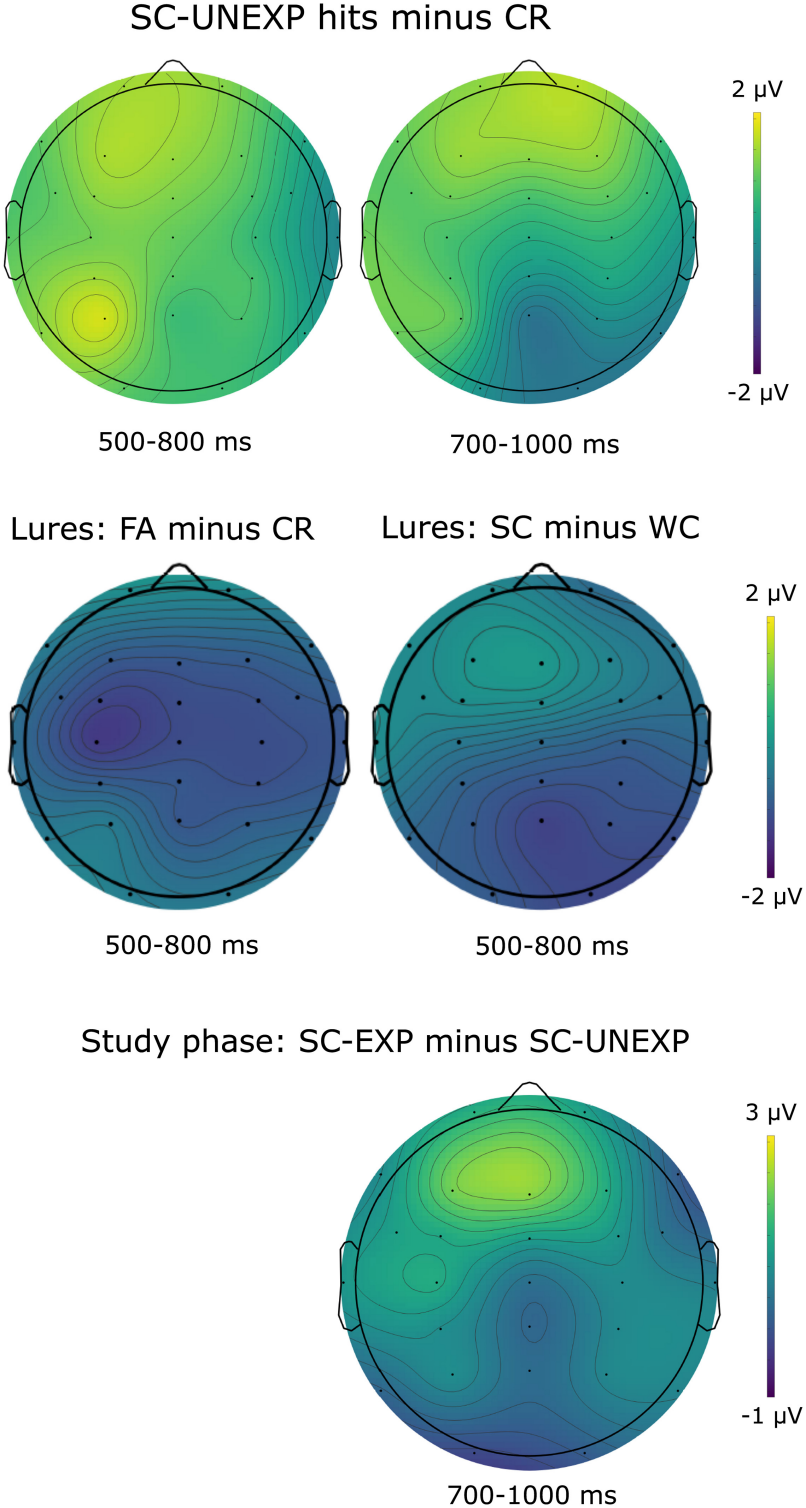
Topographical distributions of observed effects. Top: differences between SC-UNEXP hits and correct rejections during the test phase. Middle: left – differences between false alarms and correct rejections for expected lures in the test phase. Right – differences between expected lures from strong constraint (SC) versus weak constraint (WC) sentences. Bottom: differences between expected and unexpected completions of SC sentences during the study phase.

Regarding late parietal old/new effects, mean amplitudes between 500 and 800 ms at posterior electrodes were analyzed. As predicted, ERPs to hits elicited by SC-UNEXP words (*M* = 3.39 μV, SEM = 0.77) were associated with more positive mean amplitudes than those elicited by WC-UNEXP words (*M* = 2.30 μV, SEM = 0.83), *t*(35) = 3.02, *p* < 0.01 (one-tailed), *d* = 0.22. The analysis of old/new effects in each condition revealed a significant old/new effect only for SC-UNEXP hits, *t*(35) = 2.53, *p* < 0.01, *d* = 0.42, while other conditions did not exhibit significance (all *p*-values > 0.02). The (left) parietal distribution of this effects is illustrated in Figure 2.

In addition to the parietal old/new effect SC-UNEXP hits also elicited a positive slow wave, with a frontal scalp topography, which began around 400 ms post-stimulus and continued until the end of the epoch. This unexpected slow wave effect resembles the late frontal positivity which we found when the same unexpected words were presented as sentence endings of the same highly constraining sentences (Höltje and Mecklinger, 2022). To assess the statistical reliability of this unexpected effect, we conducted a repeated-measures ANOVA on mean amplitudes between 700 and 1000 ms at electrodes Fp1, Fp2, F3, Fz, and F4, with Item Status as the within-subjects factor. This analysis employed a time window and electrode configuration similar to the one used for examining the late frontal positivity in our previous study (Höltje and Mecklinger, 2022). The effect of Item Status was significant, *F*(4, 140) = 2.46, *p* < 0.05, *η*_p_^2^ = 0.07. Subsequent dependent *t*-tests confirmed that mean amplitudes were more positive for SC-UNEXP hits (*M* = 3.35 μV, SEM = 0.63) compared to SC-EXP hits (*M* = 2.30 μV, SEM = 0.56), *t*(35) = 2.69, *p* < 0.05, *d* = 0.29, whereas the difference between WC-EXP and WC-UNEXP hits was nonsignificant, *t*(35) = 0.70, *p* = 0.49, *d* = 0.10. Additionally, SC-UNEXP hits were also associated with more positive amplitudes than correct rejections to new words (*M* = 1.91 μV, SEM = 0.47), *t*(35) = 2.80, *p* < 0.01, *d* = 0.42. The topographic distribution of the late frontal positivity (SC-UNEXP vs. CR) is illustrated in Figure 2 (upper part). Thus, the successful retrieval of unexpected words that were preceded by strongly constraining sentence contexts during encoding elicited a frontal positivity which is functionally and spatiotemporally similar to a frontal positivity, typically observed during the encoding of unexpected words in strongly constraining sentence contexts (Federmeier et al., 2007; Höltje et al., 2019; Höltje and Mecklinger, 2022; Kuperberg et al., 2019; Ness and Meltzer-Asscher, 2018a; Stone et al., 2023).

#### 3.2.2 Expected lures: false alarms vs. correct rejections

**FIGURE 3:**
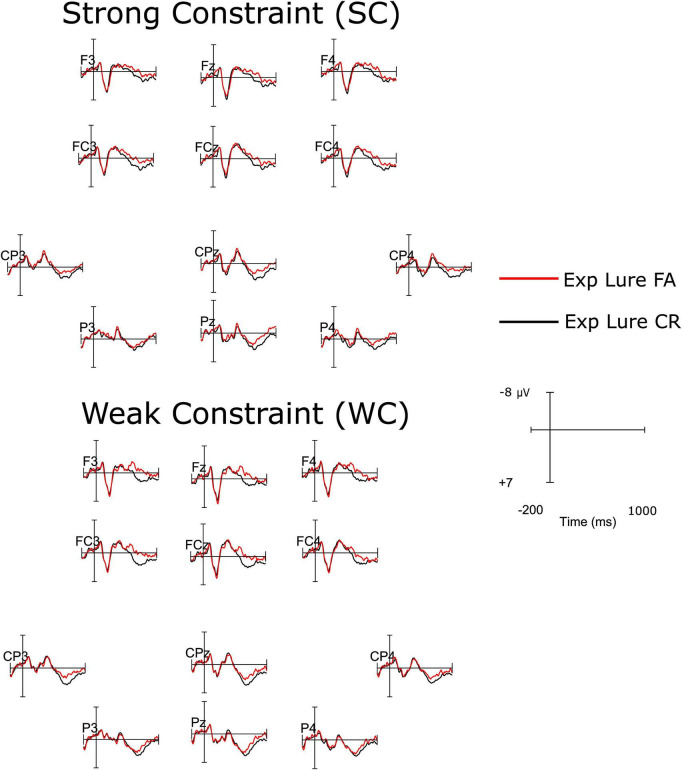
Event-related potentials (ERP) waveforms elicited at electrodes of the anterior (F3, Fz, F4, FC3, FCz, FC4) and posterior (CP3, CPz, CP4, P3, Pz, P4) electrode clusters by the onset of lure words in the recognition memory test. Top half: words encoded in strong constraint (SC) sentences, bottom half: words encoded in weak constraint (WC) sentences. The alignment of waveforms corresponds to the approximate topographical locations of electrodes over the scalp.

Figure 3 illustrates ERPs elicited by expected lures that were either falsely identified as “old” (false alarms) or correctly rejected during the recognition memory test. We explored whether memory decisions regarding expected lures influenced mean amplitudes in the N400 time window and a later time window at frontal electrodes. Mean amplitudes from 300 to 500 ms at posterior electrodes and from 500 to 800 ms at anterior and posterior electrodes were assessed using two repeated-measures ANOVAs, considering the factors Constraint (SC, WC) and Memory (correct rejections, false alarms).

In the 300–500 ms time window, the main effect of Constraint was not significant, *F*(1,27) = 3.95, *p* = 0.06, *η*_p_^2^ = 0.13, nor were the main effect of Memory (*F* < 1) and the Constraint by Memory interaction, *F*(1,27) = 1.20, *p* = 0.28, *η*_p_^2^ = 0.04.

In the later 500–800 ms time window at anterior electrodes, the main effect of Memory was significant, *F*(1,27) = 11.46, *p* < 0.01, *η*_p_^2^ = 0.30, indicating more positive amplitudes for correct rejections (*M* = 0.92 μV, SEM = 0.62) compared to false alarms (*M* = −0.64 μV, SEM = 0.88). The main effect of Constraint and the Constraint by Memory interaction were not significant (Fs < 1). At posterior electrodes, the main effects of Constraint, *F*(1,27) = 11.17, *p* < 0.01, *η*_p_^2^ = 0.29, and Memory, *F*(1,27) = 4.29, *p* < 0.05, *η*_p_^2^ = 0.14, were significant, indicating that lure words from WC sentences (*M* = 2.67 μV, SEM = 0.82) elicited more positive mean amplitudes than those from SC sentences (*M* = 1.58 μV, SEM = 0.77), and correct rejections (*M* = 2.62 μV, SEM = 0.68) were associated with more positive mean amplitudes than false alarms (*M* = 1.63 μV, SEM = 0.93). The Constraint by Memory interaction was nonsignificant. To summarize, the analysis of ERPs to expected lures yielded memory effects in the 500–800 ms time window both at anterior and posterior electrodes. As evident from Figure 2, the effects observed in relation to lure items are broadly distributed: the difference between false alarms and correct rejections shows a left-central focus, while the difference between SC and WC lures reveals a parietal maximum.

### 3.2.3 *Post hoc* analyses of study phase

**FIGURE 4:**
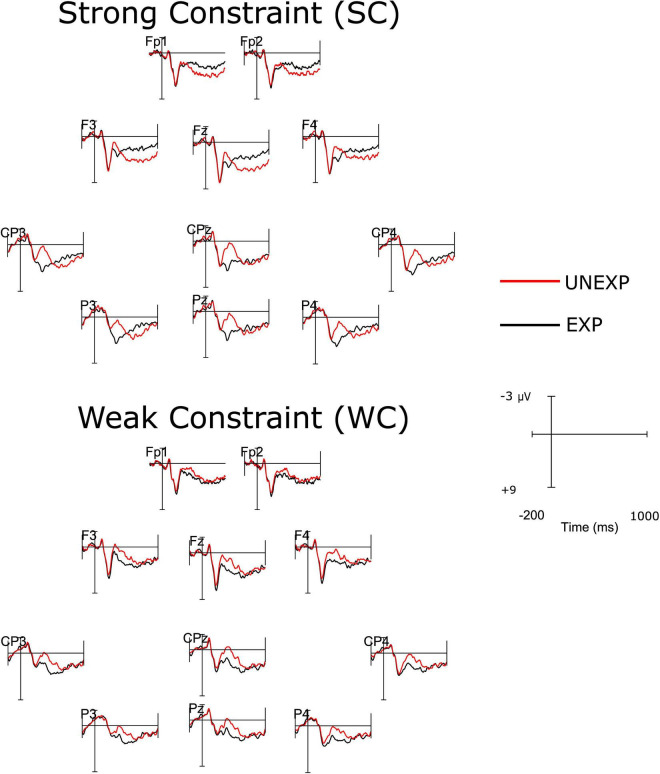
Event-related potentials (ERP) waveforms elicited at electrodes of the anterior (Fp1, Fp2, F3, Fz, F4) and posterior (CP3, CPz, CP4, P3, Pz, P4) electrode clusters by the onset of target words in the study phase. Top half: words encoded in strong constraint (SC) sentences, bottom half: words encoded in weak constraint (WC) sentences. The alignment of waveforms corresponds to the approximate topographical locations of electrodes over the scalp.

The successful retrieval of unexpected words that were preceded by strongly constraining sentence contexts during encoding elicited a frontal positivity which is functionally and spatiotemporally similar to a frontal positivity, typically observed during the encoding of unexpected words in strongly constraining sentence contexts. Given this unexpected finding we also explored whether a similar LFP is also present in the study phase of the present study. ERPs elicited by target words in the study phase are shown in Figure 4. The analysis was based on data from *n* = 34 subjects as four data sets had to be excluded due to excessive artifacts contaminating the EEG data. We analyzed mean amplitudes between 700 and 1000 ms post-stimulus at electrodes Fp1, Fp2, F3, Fz, and F4 in an ANOVA including the factors Constraint (SC, WC) and Expectedness (EXP, UNEXP). We found a significant main effect of Expectedness, *F*(1,33) = 5.20, *p* < 0.05, *η*_p_^2^ = 0.14, qualified by a significant Constraint by Expectedness interaction, *F*(1,33) = 13.62, *p* < 0.001, *η*_p_^2^ = 0.29. The main effect of Constraint turned out nonsignificant, *F*(1,33) < 1, *p* = 0.62, *η*_p_^2^ = 0.01. Subsidiary *t*-tests revealed that SC-UNEXP words were associated with more positive mean amplitudes than SC-EXP words (SC-EXP: *M* = 2.39 μV, SEM = 0.53; SC-UNEXP: *M* = 4.22 μV, SEM = 0.53; *t*(33) = 3.68, *p* < 0.001, *d* = 0.60) whereas the difference between WC-UNEXP and WC-EXP was not significant (WC-EXP: *M* = 3.31 μV, SEM = 0.50; WC-UNEXP: *M* = 2.99 μV, SEM = 0.50; *t*(33) = 1.48, *p* = 0.15, *d* = 0.11). Furthermore, SC-UNEXP words (*M* = 4.22 μV, SEM = 0.53) were associated with more positive mean amplitudes than WC-UNEXP words (*M* = 2.99 μV, SEM = 0.50), *t*(33) = 2.79, *p* < 0.01, *d* = 0.41. As evident from Figure 2 (bottom), the frontal positivity associated with the processing of SC-UNEXP words during the study phase displays a pronounced frontal distribution.

## 4 Discussion

This study investigated the mnemonic consequences and neurocognitive processes associated with the retrieval of expected words confirming predictions and unexpected words that trigger expectancy mismatches. Building on our previous work (Höltje and Mecklinger, 2022), we hypothesized that predictive sentence contexts would especially facilitate the processing of expected words. We further anticipated that these contexts would activate schemas, leading to the formation of more stable, semantically elaborated memory traces and superior recognition of expected words. Regarding the unexpected words, we predicted that a memory-enhancing effect of prediction errors on memory would be present under the short retention delay conditions in the present study.

As expected, hit rates followed a U-shaped function: They were similarly high for SC-EXP and SC-UNEXP words, slightly lower for WC-EXP words, and significantly lower for WC-UNEXP words. This pattern suggests that strongly constraining (SC) and even weakly constraining (WC) contexts provided sufficient schema support to improve the encoding of expected words, making them more easily retrievable. In contrast, unexpected words showed high hit rates only when they were encoded as the completions of SC sentences, highlighting the memory-enhancing effect of prediction errors. Overall, these findings provide behavioral evidence for memory enhancement for both, expected words that confirm predictions and unexpected words that challenge expectations.

Theoretical frameworks propose that prediction errors caused by expectancy mismatches enhance learning by capturing attention, leading to deeper encoding of unexpected events (Butterfield and Metcalfe, 2006; Fazio and Marsh, 2009; Henson and Gagnepain, 2010; Kuperberg and Jaeger, 2016). Accordingly, we hypothesized that unexpected words would benefit from a memory advantage due to the prediction errors they evoke, with this effect depending on the strength of the sentence context’s predictions. Consistent with this hypothesis, memory performance was highest for both strongly expected (SC-EXP) and strongly unexpected (SC-UNEXP) words, with both conditions yielding nearly identical hit rates. This finding supports the idea that both highly expected and highly unexpected words are more memorable than words for which no strong expectations were generated (Greve et al., 2019).

Our result that both SC-EXP and SC-UNEXP words were remembered equally well contrasts with the findings of our previous study (Höltje and Mecklinger, 2022), where SC-EXP words were recognized more accurately than SC-UNEXP words. A key difference between the two studies was the retention interval between the learning and test phases–1 day in Höltje and Mecklinger (2022) versus 12 min in the current study. Taken together, the results from both studies suggest that schema congruency and expectancy mismatches improve memory, but on different timescales. Schema-congruent memories remain stable and are robust even after a 24-h delay, while enhanced memory for expectancy mismatches is short-lived and only observed after a short retention interval like in the present study. These findings align with previous research by Tompary et al. (2020) and van Kesteren et al. (2013), supporting schema consolidation theories. These theories propose that schema-congruent memories are preferentially and rapidly consolidated (McKenzie and Eichenbaum, 2011; van Kesteren et al., 2012; Wang and Morris, 2010), leading to a strengthening of the influence of schema congruency on memory performance over time. Our findings are also consistent with the view that forgetting of hippocampal memories is driven by a relatively fast decay process whereas extra-hippocampal memories are unaffected by this type of forgetting (e.g., Sadeh et al., 2014).

Given that we found a beneficial effect of prediction errors on memory performance at shorter time scales, we were interested in firstly, replicating this result pattern in a separate experiment with a short retention interval and secondly, testing whether distinctiveness plays a role in prediction error-driven learning (e.g., Reggev et al., 2018). We therefore conducted a separate behavioral study and found results consistent with the EEG experiment reported here. Specifically, we observed memory-enhancing effects for both expected words that confirm predictions and unexpected words that challenge expectations. Notably, this pattern emerged under both low and high distinctiveness conditions.^1^ We conclude that distinctiveness does not seem to play a role for unexpectancy-driven learning in our paradigm.

In this study, we analyzed event-related potentials (ERPs) recorded during memory retrieval to investigate how the strength of schema support provided by sentence contexts during encoding affects the recognition of target words. Our hypotheses predicted that correctly identified expected “old” words would elicit early frontal and late parietal old/new effects, reflecting the contribution of both, relative familiarity and recollection, to schema supported memory retrieval. However, we found no evidence for the early frontal old/new effects typically associated with relative familiarity. Instead, a late parietal old/new effect was observed for SC-UNEXP words, which likely triggered strong expectancy violations during encoding. This pattern is consistent with our behavioral results, where SC-UNEXP words were remembered better than WC-UNEXP words, which neither aligned with an activated schema nor generated substantial prediction errors during encoding. Our results align with those of Hubbard and Federmeier (2024), who found that unexpected but plausible words elicited a late positive complex (LPC). These findings suggest that words causing significant expectancy violations during reading are subsequently more often recognized on the basis of recollection–a slow and controlled process by which qualitative details from a study episode are recovered and which depends on hippocampal integrity (Eichenbaum et al., 2007; Yonelinas, 2002). This supports neurocognitive models proposing that prediction errors enhance memory through hippocampal involvement (Henson and Gagnepain, 2010; van Kesteren et al., 2012).

Interestingly, we did not observe any ERP retrieval effects accompanying the congruency effect on memory performance (i.e., better memory for expected words). Of note, in our earlier study (Höltje and Mecklinger, 2022), we found larger parietal subsequent memory effects (SMEs) during encoding for expected versus unexpected words, presumably reflecting item-specific encoding that enhances their distinctiveness in memory. It is possible that the distinctive memory representation of expected words, formed through extensive encoding, enabled their relatively effortless retrieval, resulting in small (nonsignificant) familiarity effects. In contrast, strongly unexpected words appeared to initiate more robust recollective processing, as indicated by the late parietal old/new effects in the current study.

The retrieval of SC-UNEXP words additionally gave rise to a positive slow wave which resembles the late frontal positivity (LFP) associated with the processing of unexpected but plausible words in highly constraining sentences (Federmeier et al., 2007; Höltje and Mecklinger, 2022; Kuperberg et al., 2020; Ness and Meltzer-Asscher, 2018a). Confirming this latter view, our *post hoc* analyses of the study phase data revealed a similar LFP to unexpected endings of highly constraining sentences. We did not anticipate this activity in the test phase since the recognition test presented words in isolation, without any sentence context allowing expectancies to build up, and the LFP is typically seen when words trigger an expectancy mismatch during language comprehension and the suppression of a strongly predicted word is required.

It is conceivable that the retrieval of unexpected words in the current study involved a similar suppression process. Strongly constraining sentences promote early binding between words and their context without requiring extensive encoding efforts (Höltje and Mecklinger, 2022). This may occur if highly predictive contexts enhance semantic integration and relational binding during encoding, resulting in memory traces that are more readily accessible later (Staresina et al., 2009). Retrieving an unexpected word may trigger the reactivation of the original sentence context, making it necessary to suppress the predicted but not presented word again. This suppression would help guide memory decisions, especially when participants are required to reject expected lures as “new,” as in this study. This functional interpretation of the LFP is also supported by our finding that this type of activity was exclusively linked to hits, but not to correct rejections, suggesting that this signal elicited by processing strong prediction errors might have been used to guide memory decisions.

One of the key objectives of this study was to further investigate the fate of words that were expected but never actually encountered during the study phase, and which were later presented as lures in the recognition memory test. Consistent with findings from Hubbard et al. (2019), we hypothesized that predicted but unpresented words are pre-activated during the processing of sentence contexts in the study phase and might remain in a state of increased pre-activation until the test phase. This pre-activation could enhance the processing fluency of expected lures during the test, leading to a higher likelihood of false positive memory decisions. If stronger schema-based predictions result in greater pre-activation, we expected lures from strongly constraining (SC) sentences to yield more false alarms than those from weakly constraining (WC) sentences. This is exactly what we found: Expected lures elicited more false alarms than unrelated new words, with SC lures producing higher false alarm rates than WC lures. This replicates our previous results (Höltje and Mecklinger, 2022) and demonstrates that while predictive processing offers memory benefits, it can also be detrimental.

Hubbard et al. (2019) proposed that false alarms to expected lures from SC sentences are driven by increased conceptual fluency due to the pre-activation of predicted words, as evidenced by attenuated N400 responses during retrieval in their study. In contrast, we did neither find N400 attenuation effects nor evidence for relative familiarity (early frontal old/new effects) for SC lures, although they provoked higher false alarm rates. Rather, we found that correctly rejected lures elicited more positive-going ERPs than false alarms in the later 500–800 ms interval. This suggests that, in our study, induced by the length of the retention interval, different processes mediated the false alarms to SC lures compared to those observed by Hubbard et al. (2019). It has been proposed that strong lexical predictions lead to an updating of the sentence context with the predicted word in working memory (Lau et al., 2013; Ness and Meltzer-Asscher, 2018b). It is conceivable that in our experiment, the 1-s delay between sentence contexts and target words during encoding strengthened predictions and updating of sentence representations. If these updated sentence representations had not been sufficiently revised when the predicted word was disconfirmed, the predicted but unpresented word might have persisted in memory. In the test phase, the processing of words that had been strongly expected but not actually encoded during the study phase could have elicited recollective processing as reflected in the positive-going ERPs to lures between 500 and 800 ms at posterior electrodes. The lingering of the updated sentence representation could have led the participants to adopt a recall-to-reject strategy (“I remember that I strongly expected this word in the study phase, but that surprisingly, it was not presented, so I am going to reject it”).

In the Hubbard et al. (2019) study, correct rejections of WC lures were associated with a broadly distributed positive slow wave between 500 and 1000 ms, resembling the right frontal old/new effect, which has been linked to decision-making and post-retrieval monitoring in other recognition memory studies (Cruse and Wilding, 2009; Hayama et al., 2008; Rosburg et al., 2011). In our study, correctly rejected lures also elicited more positive-going ERPs than false alarms between 500 and 800 ms at frontal electrode sites. This effect, however, occurred earlier than the typical right frontal old/new effect, which usually appears not before 800 ms. The timing of the correct rejections > false alarms ERP effect which was also present at posterior electrodes rather resembles the late parietal old/new effect associated with recollective processing, which suggests that our participants may have employed a recall-to-reject strategy for both SC and WC lures (as discussed above).

In summary, our results highlight that sentence context strength has multiple behavioral and electrophysiological effects on memory retrieval. Behaviorally, strongly expected and highly unexpected words were more likely to be recognized, whereas memory for moderately expected words was poorer. Electrophysiologically, retrieval of highly unexpected words gave rise to a late parietal old/new effect, indicating recollective processing, and a late frontal positivity, potentially reflecting the reactivation of inhibitory control processes from the prior encoding phase. Additionally, participants showed a higher tendency to falsely recognize highly predictable but unpresented words as “old.” Despite greater behavioral challenges in rejecting SC lures, our ERP findings indicate that the rejection of both strongly and weakly expected lures relied on similar neural mechanisms, namely recollective processing supporting a recall-to-reject strategy.

## Data Availability

The raw data supporting the conclusions of this article will be made available by the authors, without undue reservation.

## Footnotes

1 To investigate whether the distinctiveness of contextually unexpected words influenced memory performance, we conducted a separate behavioral experiment with *N* = 45 participants, divided into two groups (50/50, 80/20). In the 50/50 group, participants encoded 50 words from each experimental condition (SC-EXP, SC-UNEXP, WC-EXP, WC-UNEXP), following a procedure similar to the EEG experiment. The study and test phases took place on the same day, similar to the EEG experiment reported in the present study. In the 80/20 group, participants encoded 80 SC-EXP and 80 WC-EXP words, but only 20 SC-UNEXP and 20 WC-UNEXP words, making unexpected words less frequent and thus more distinctive. If distinctiveness enhances memory for unexpected words, we would expect similar or better memory performance for unexpected words compared to expected ones in the 80/20 group. However, the results showed that increasing the distinctiveness of unexpected words did not impact memory performance, as indicated by a nonsignificant group effect. Instead, the overall memory performance was influenced by an interaction between sentence constraint and expectedness. Specifically, better memory for expected over unexpected words was observed in weakly constraining sentences, but not in strongly constraining ones. This pattern closely mirrors the findings of the present study, suggesting that the memory advantage for expected words is robust, even when the distinctiveness of unexpected words is increased.
